# Histone deacetylase inhibitors restore normal hippocampal synaptic plasticity and seizure threshold in a mouse model of Tuberous Sclerosis Complex

**DOI:** 10.1038/s41598-019-41744-7

**Published:** 2019-03-27

**Authors:** Trina Basu, Kenneth J. O’Riordan, Barry A. Schoenike, Nadia N. Khan, Eli P. Wallace, Genesis Rodriguez, Rama K. Maganti, Avtar Roopra

**Affiliations:** 1 Department of Neuroscience, University of Wisconsin-Madison, Madison, Wisconsin United States of America; 2 Neuroscience Training Program, University of Wisconsin-Madison, Madison, Wisconsin United States of America; 3 Graduate Program in Cellular and Molecular Biology, University of Wisconsin-Madison, Madison, Wisconsin United States of America; 4 Cellular and Molecular Pathology Graduate Program, University of Wisconsin-Madison, Madison, Wisconsin United States of America; 5 Department of Neurology, University of Wisconsin-Madison, Madison, Wisconsin United States of America

## Abstract

Abnormal synaptic plasticity has been implicated in several neurological disorders including epilepsy, dementia and Autism Spectrum Disorder (ASD). Tuberous Sclerosis Complex (TSC) is an autosomal dominant genetic disorder that manifests with seizures, autism, and cognitive deficits. The abnormal intracellular signaling underlying TSC has been the focus of many studies. However, nothing is known about the role of histone modifications in contributing to the neurological manifestations in TSC. Dynamic regulation of chromatin structure via post translational modification of histone tails has been implicated in learning, memory and synaptic plasticity. Histone acetylation and associated gene activation plays a key role in plasticity and so we asked whether histone acetylation might be dysregulated in TSC. In this study, we report a general reduction in hippocampal histone H3 acetylation levels in a mouse model of TSC2. Pharmacological inhibition of Histone Deacetylase (HDAC) activity restores histone H3 acetylation levels and ameliorates the aberrant plasticity in TSC2^+/−^ mice. We describe a novel seizure phenotype in TSC2^+/−^ mice that is also normalized with HDAC inhibitors (HDACis). The results from this study suggest an unanticipated role for chromatin modification in TSC and may inform novel therapeutic strategies for TSC patients.

## Introduction

Synaptic plasticity underlies mechanisms for encoding new information and forming long term memory in the mammalian hippocampus^1–4^. Aberrations in acquiring or maintaining synaptic plasticity have been linked to cognitive deficits, intellectual disability, epilepsy and autism spectrum disorder (ASD)^5–8^.

Tuberous Sclerosis Complex (TSC) is an autosomal dominant, multisystem spectrum disorder that affects approximately 1 in 6,000 people. The condition is characterized by formation of benign growths that most commonly develop in the brain, kidney, heart, lungs, eyes and skin. Patients with TSC display developmental delays, cognitive defects and autism. In addition, over 85% of patients develop epilepsy within the first year of life^9,10^.

TSC is caused by a loss of function mutation in either the *TSC1* or *TSC2* genes^11,12^. The TSC1 (hamartin) and TSC2 (tuberin) proteins heterodimerize to form a GTPase activating protein (GAP) complex which inhibits the mammalian Target of Rapamycin Complex 1 (mTORC1) via negative regulation of the GTP binding protein, Rho enriched in the brain (Rheb)^13^. In the brain, mTORC1 signaling pathway is a critical kinase hub that regulates post-synaptic protein translation to influence synaptogenesis, dendritic and axonal growth, and activity dependent synaptic plasticity^11,14–17^. A mutation in either *TSC1* or *TSC2* results in altered mTORC1 signaling and aberrant hippocampal synaptic plasticity, impairments in learning and memory, epilepsy, and autism-like behavioral phenotypes^18–22^.

Previous reports on TSC2^+/−^ mice have focused on either the constitutively active mTORC1^19,20,22,23^ or the aberrantly increased mitogen activating protein kinase (MAPK) signaling^24^ to restore normal synaptic plasticity. However, nothing is known about the role of histone modifications in TSC even though post translational modifications (PTMs) of histone tails have been shown to govern long term memory formation and Long Term Potentiation (LTP)^25–28^.

Pharmacological inhibition or genetic knock down of Histone Deacetylases (HDACs) in rodents enhances cognition *in vivo* and synaptic plasticity *in vitro*^29–34^. In addition, several studies have shown an increase in histone H3 acetylation levels in the rodent hippocampus after performance in hippocampal dependent behavioral paradigms^35–37^. In combination, these studies demonstrate that increased acetylation enhances synaptic plasticity and cognition.

Pharmacological inhibitors of class I/II HDACs have also been shown to augment LTP responses in acute hippocampal slice preparations. In particular, bath application of the class I/II HDAC inhibitor (HDACi), Trichostatin A (TSA), induces a long lasting LTP under a stimulation paradigm that normally elicits short term potentiation (STP) in adult wild type (WT) hippocampal slices^29,35,38^.

Interestingly, we and others have reported that adult TSC2^+/−^ mice exhibit an enhanced LTP under stimulation protocols that normally elicit STP^20^. Thus, we hypothesized that TSC2^+/−^ mice may have decreased HDAC activity and increased histone acetylation levels. However, contrary to this hypothesis, we find that the TSC2^+/−^ mouse hippocampus exhibits decreased acetylation levels. Because HDACs work in balance with Histone Acetyltransferase (HAT) activity, the reduced acetylation levels in the TSC2^+/−^ mice suggests that there may be a disparity in the ratio of HDAC to HAT function. In this study, we show that pharmacological attenuation of HDAC activity in TSC2^+/−^ mice restores synaptic plasticity and normalizes a novel seizure threshold phenotype to resemble a WT response.

## Results

### TSC2^+/−^ mouse hippocampi exhibit reduced global histone acetylation levels

**Figure 1:**
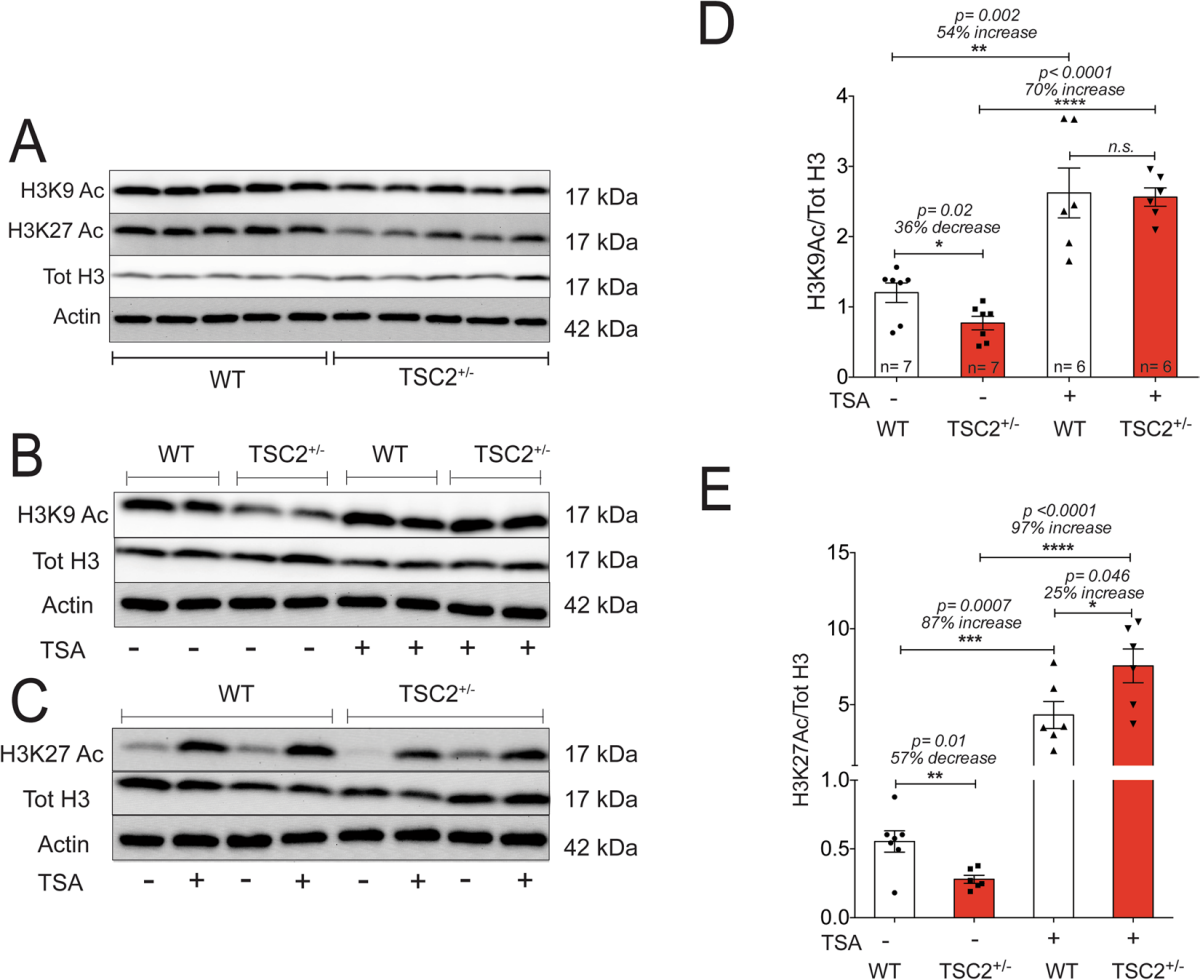
TSC2^+/−^ mouse hippocampi exhibit decreased global histone acetylation levels. (**A)** Representative cropped western blots of acute hippocampal slices acquired from adult WT and TSC2^+/−^ mice (n = 5 animals per genotype). Each lane represents hippocampal lysate from a single animal. Hippocampal slices were harvested following incubation in artificial cerebrospinal fluid (ACSF) for 4 hours. Representative cropped western blots depicting H3K9 Ac protein levels (**B**) and H3K27 Ac (**C**) in acute hippocampal slices from adult WT and TSC2^+/−^ animals harvested either in the presence or absence of the HDAC inhibitor, TSA (1.65 µM). Quantification of H3K9 Ac (**D**) and H3K27 Ac (**E**) protein levels from acutely harvested hippocampal slices from adult WT and TSC2^+/−^ mice treated with or without TSA.

We originally posited that the enhanced LTP observed in the TSC2^+/−^ mouse model would be due to decreased HDAC function and increased acetylation levels. To test this hypothesis, we collected acute hippocampal slices from adult WT and TSC2^+/−^ mice (6–8 weeks old) and analyzed protein levels from the homogenized lysates. In contrast to our hypothesis, TSC2^+/−^ mice exhibit decreased acetylation levels at lysine residues 9 and 27 of histone H3 (H3K9Ac and H3K27Ac) compared to WT littermates (Fig. 1A). The global reduction of histone acetylation levels suggests an imbalance between histone deacetylase and acetylase activity in the TSC2^+/−^ mouse brain.

### HDAC inhibition evokes STP in adult TSC2^+/−^ mice

**Figure 2:**
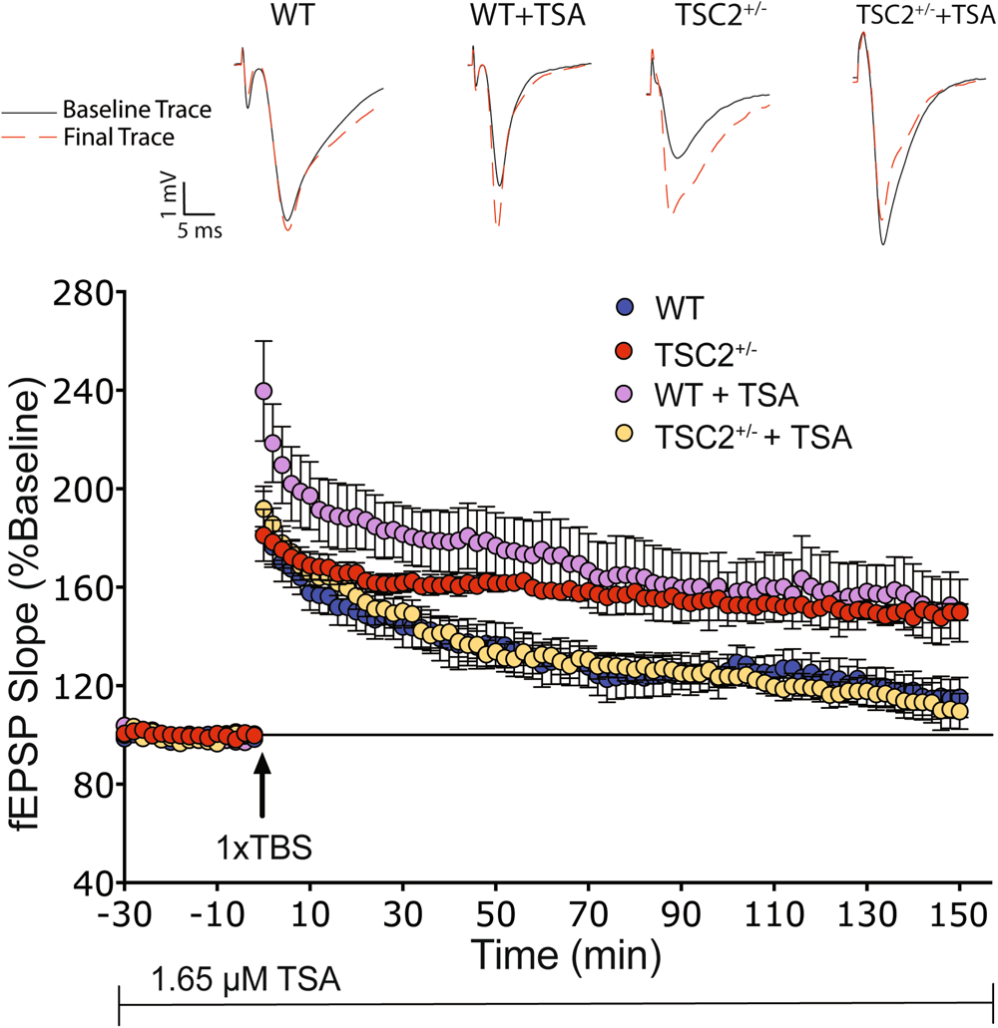
HDAC inhibition restores WT-like STP in response to 1 × TBS in adult TSC2^+/−^ mice. A 1 × TBS elicits STP in adult WT hippocampal slices (shown in blue; n = 11 slices from 5 mice), while it provokes long lasting LTP in adult TSC2^+/−^ mice (shown in red; n = 9 slices from 5 mice; two-way ANOVA: *F*(1,17) = 10.45, *p* = 0.0049). TSC2^+/−^ slices incubated in TSA (1.65 µM) exhibited STP that was indistinguishable from untreated WT slices (n = 7 slices from 5 mice; two-way ANOVA: *F*(1,15) = 0.0007718, *p* = 0.9782). WT slices treated with TSA display long lasting LTP similar to that seen in TSC2^+/−^ hippocampal slices (shown in purple; n = 6 slices from 6 mice; two-way ANOVA: *F*(1.13) = 1.557, *p* = 0.2350). TSA was introduced to the slices 60 minutes prior to 1 × TBS and was kept on for the duration of the experiment.

We asked whether the potential imbalance of HDAC and HAT activity contributes to plasticity alterations observed in rodent models of TSC. We tested whether HDAC inhibitors (HDACis) would restore histone acetylation levels to WT and ameliorate the abnormal LTP phenotype seen in TSC2^+/−^ mice. As reported by others^39^, we find that a single theta burst (1 × TBS) elicits STP in WT mice (Fig. 2, shown in blue). The same stimulation results in a stable LTP in TSC2^+/−^ mice (Fig. 2, shown in red), similar to the phenotype exhibited by TSC2^+/−^ mice in response to a sub-threshold 1 × 100 Hz stimulation as reported by Ehninger *et al*.^20^. Surprisingly, in the presence of 1.65 µM Trichostatin A (a class I/II HDACi), 1 × TBS elicited STP in TSC2^+/−^ slices that is indistinguishable from the 1 × TBS/no drug WT response (Fig. 2, shown in yellow). As described by others^29,35^, 1 × TBS + TSA resulted in a robust LTP in WT slices (Fig. 2, shown in purple).

This observation suggests that inhibiting HDAC activity produces an LTP in adult TSC2^+/−^ hippocampal slices that resembles the untreated WT response. Furthermore, HDAC inhibition has opposing effects in the response elicited by a 1 × TBS paradigm in WT and TSC2^+/−^mice.

### HDAC inhibition restores normal mGluR-LTD in juvenile TSC2^+/−^ mice

**Figure 3:**
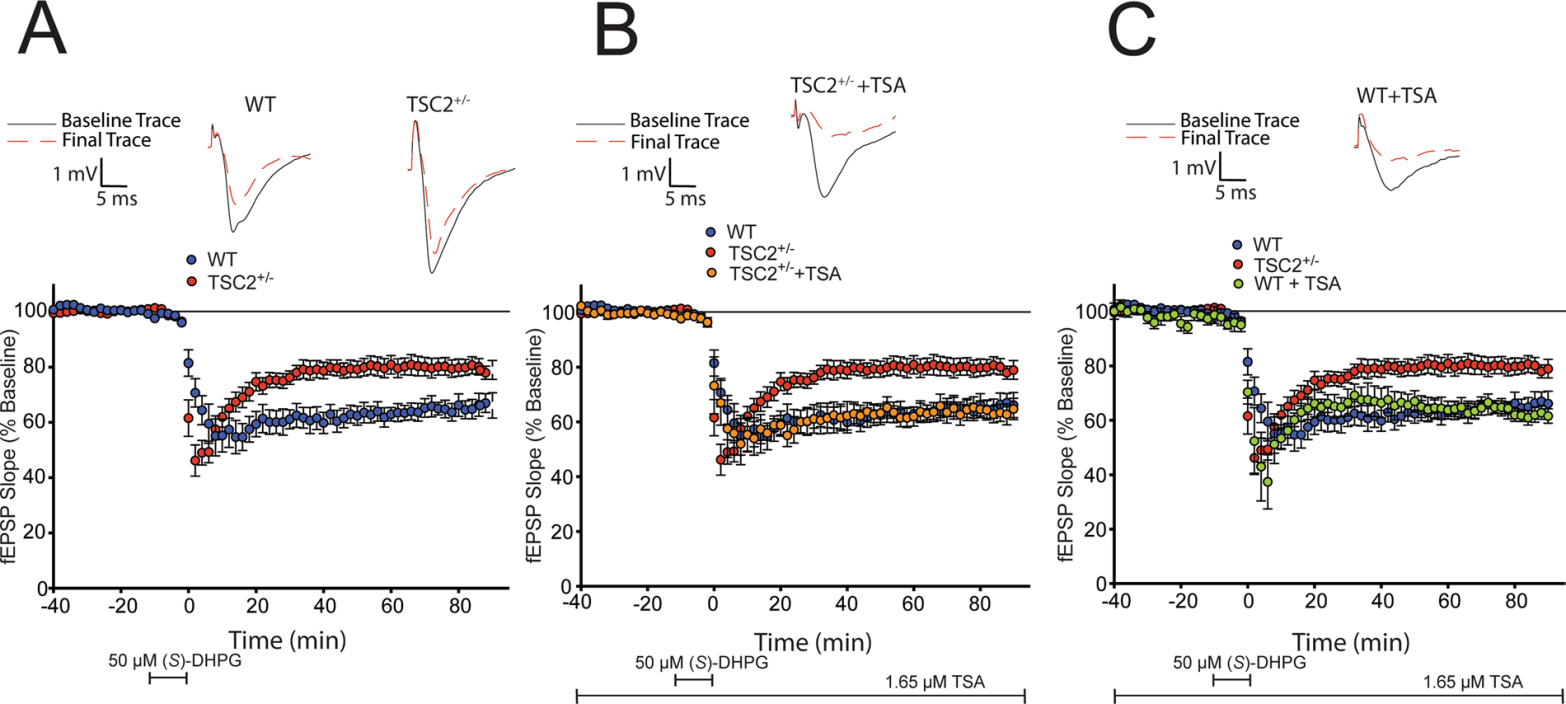
HDAC inhibition restores mGluR-LTD in juvenile TSC2^+/−^ mice to mimic a juvenile WT response. (**A)** Acute hippocampal slices obtained from juvenile TSC2^+/−^ mice (shown in red; n = 14 slices from 6 mice) exhibit a decreased mGluR-LTD magnitude compared to age matched littermate WT mice (shown in blue; n = 12 slices from 5 mice; two-way ANOVA: *F*(1,19) = 7.079, *p* < 0.0001). (**B)** Juvenile TSC2^+/−^ slices treated with TSA (1.65 µM) display an mGluR-LTD that is indistinguishable from untreated juvenile WT slices (shown in orange; n = 8 slices from 4 mice; two-way ANOVA: *F*(1,13) = 0.7724, *p* = 0.7724). (**C)** Juvenile WT slices treated with TSA do not exhibit altered mGluR-LTD magnitude (shown in green; n = 5 slices from 5 mice); two-way ANOVA: *F*(1,10) = 0.02719, *p* = 0.8723).

We went on to explore the effects of reducing HDAC activity on other synaptic plasticity alterations that have been characterized in the TSC2^+/−^ mouse model. We and others have shown that juvenile (p21–p24) TSC2^+/−^ mice display a reduced mGluR-LTD magnitude compared to age matched litter mate WT mice^19,24^. Induction of mGluR-LTD in slices using the group I mGluR agonist, (*S*)-3, 5-dihydroxyphenylglycine (DHPG; 50 µM for 10 minutes) produces a reduced mGluR-LTD magnitude in juvenile TSC2^+/−^ mice compared to age matched WT controls (Fig. 3A).

We incubated juvenile WT and TSC2^+/−^ slices with TSA (1.65 µM) for 1 hour prior to the introduction of (*S*)-DHPG. In the presence of TSA, juvenile TSC2^+/−^ hippocampal slices displayed an LTD magnitude that was indistinguishable from age matched WT slices (Fig. 3B, shown in orange). Juvenile WT hippocampal slices were unaffected by TSA treatment (Fig. 3C, shown in green). Thus, HDAC inhibition restores a normal mGluR-LTD magnitude in TSC2^+/−^ slices under conditions that do not affect WT slices.

### HDAC inhibition produces a normal, mTORC1 dependent mGluR-LTD in adult TSC2^+/−^ mice

**Figure 4:**
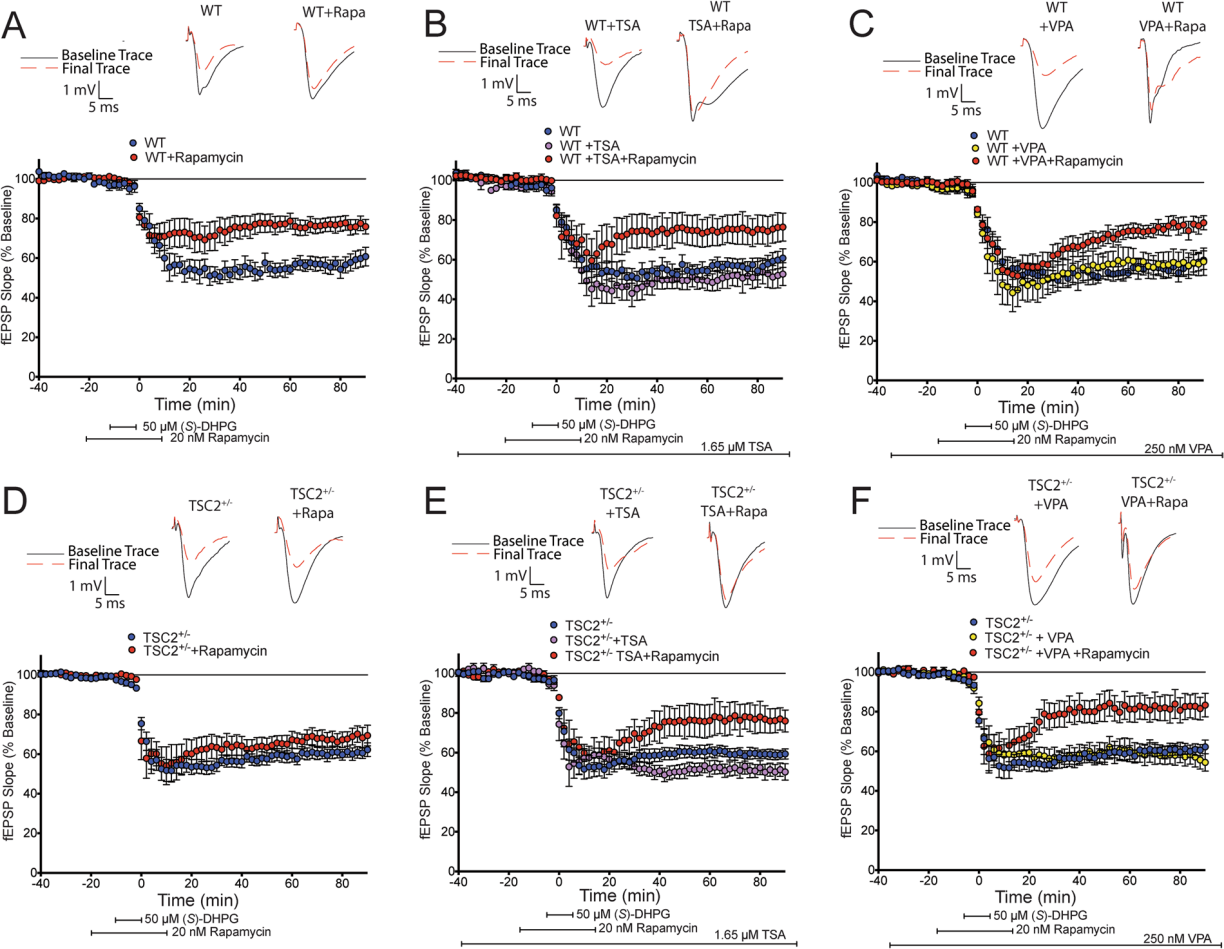
HDAC inhibition restores mTORC1 dependent mGluR-LTD in adult TSC2^+/−^ mice. (**A)** Adult WT mouse hippocampal slices (6–8 weeks old) exhibit LTD with (*S*)-DHPG (50 µM for 10 minutes; shown in blue, n = 5 slices from 4 mice) that is reduced in magnitude with rapamycin (20 nM for 20 minutes; shown in red, n = 8 slices from 4 mice; two-way ANOVA: *F*(1,15) = 7.375, *p* = 0.0159).The addition of the class I HDAC inhibitors, TSA (**B**, 1.65 µM; shown in purple, n = 5 slices from 4 mice) and VPA (**C**, 250 nM; shown in yellow, n = 6 slices from 4 mice) does not alter the acquisition of mGluR-LTD (**B**: two-way ANOVA: *F*(1.9) = 2.081, *p* = 0.1831. **C**: two-way ANOVA: *F*(1,11) = 0.1725, *p* = 0.6858) nor does it affect rapamycin sensitivity in adult WT hippocampal slices (shown in red for both; **B**: n = 7 slices from 4 mice; two-way ANOVA: *F*(1,11) 12.27, *p* = 0.0050; **C**: n = 7 slices from 5 mice; two-way ANOVA: *F*(1,10) = 9.629, *p* = 0.0112). **D)** Adult TSC2^+/−^ mice exhibit LTD with (*S*)-DHPG (shown in blue, n = 8 slices from 4 mice) that is not sensitive to rapamycin (shown in red, n = 9 slices from 5 mice). **E)** Acquisition of mGluR-LTD is not altered in TSC2^+/−^ slices treated with TSA (untreated slices shown in blue, n = 8 slices from 5 mice; TSA treated slices shown in purple n = 8 slices from 5 mice; two way ANOVA: *F*(1,18) = 1.271, *p* = 0.2743). TSC2^+/−^ slices treated with TSA exhibit rapamycin sensitivity (shown in red, n = 6 slices from 5 mice; two-way ANOVA: *F*(1,12) = 7.566, *p* = 0.0176). **F)** VPA also restores rapamycin sensitivity in adult TSC2^+/−^ slices (shown in red, n = 9 slices from 5 mice) and produces a decreased magnitude compared to slices treated with only VPA (shown in yellow, n = 7 slices from 5 mice; two-way ANOVA: *F*(1,14) = 6.054, *p* = 0.0275).

Induction of mGluR-LTD in the CA1 region of the hippocampus is dependent on mTORC1 mediated signaling and protein synthesis^40^. In hippocampal slices from adult WT animals, (*S*)-DHPG (50 µM for 10 minutes) produces a rapamycin sensitive LTD (Fig. 4A). Similar to results we have shown before^24^, adult TSC2^+/−^ slices display a mechanistically distinct LTD that is rapamycin insensitive (i.e., mTORC1 independent) despite a magnitude that is indistinguishable from adult WT slices (Fig. 4D).

To test whether HDAC inhibition would restore mTORC1 dependent LTD in TSC slices, we induced LTD in adult hippocampal slices in the presence of TSA. While bath application of TSA alone did not alter mGluR-LTD, it restored rapamycin sensitivity in adult TSC2^+/−^ hippocampal slices (Fig. 4E, shown in red). Neither (*S*)-DHPG mGluR-LTD nor sensitivity to rapamycin were altered in WT hippocampal slices exposed to TSA (Fig. 4B). This suggests that attenuation of HDAC function can restore mTORC1 dependent mGluR-LTD in the TSC2^+/−^ mouse hippocampus.

To further test the effect of HDAC inhibition in TSC2^+/−^ mice, we repeated the mGluR-LTD experiments using a structurally distinct class I/II HDACi, valproic acid (VPA). We found that bath application of VPA (250 nM) alone did not alter mGluR-LTD acquisition in either the WT or TSC2^+/−^ hippocampal slices (Fig. 4C,F). Similar to the results seen with TSA treatment, application of VPA restored rapamycin sensitivity in acute hippocampal slices from adult TSC2^+/−^ mice (Fig. 4F) without disrupting mTORC1 dependency in adult WT hippocampal slices (Fig. 4C). Thus, two structurally distinct HDACis are able to suppress the altered LTD phenotype in TSC2^+/−^ slices.

### Juvenile TSC2^+/−^ mice display a decreased seizure threshold that is normalized with SAHA

Approximately 85% of human TSC patients exhibit seizures within the first year of life, with as many as two-thirds of the patients exhibiting intractable epilepsy^9,10,41–43^. Though TSC patients have a high risk of developing epilepsy, not all TSC rodent models exhibit an overt epilepsy phenotype^42^. Here, we report the novel finding that juvenile TSC2^+/−^ mice (p18–p21) have a reduced seizure threshold compared to age matched WT mice.

**Figure 5:**
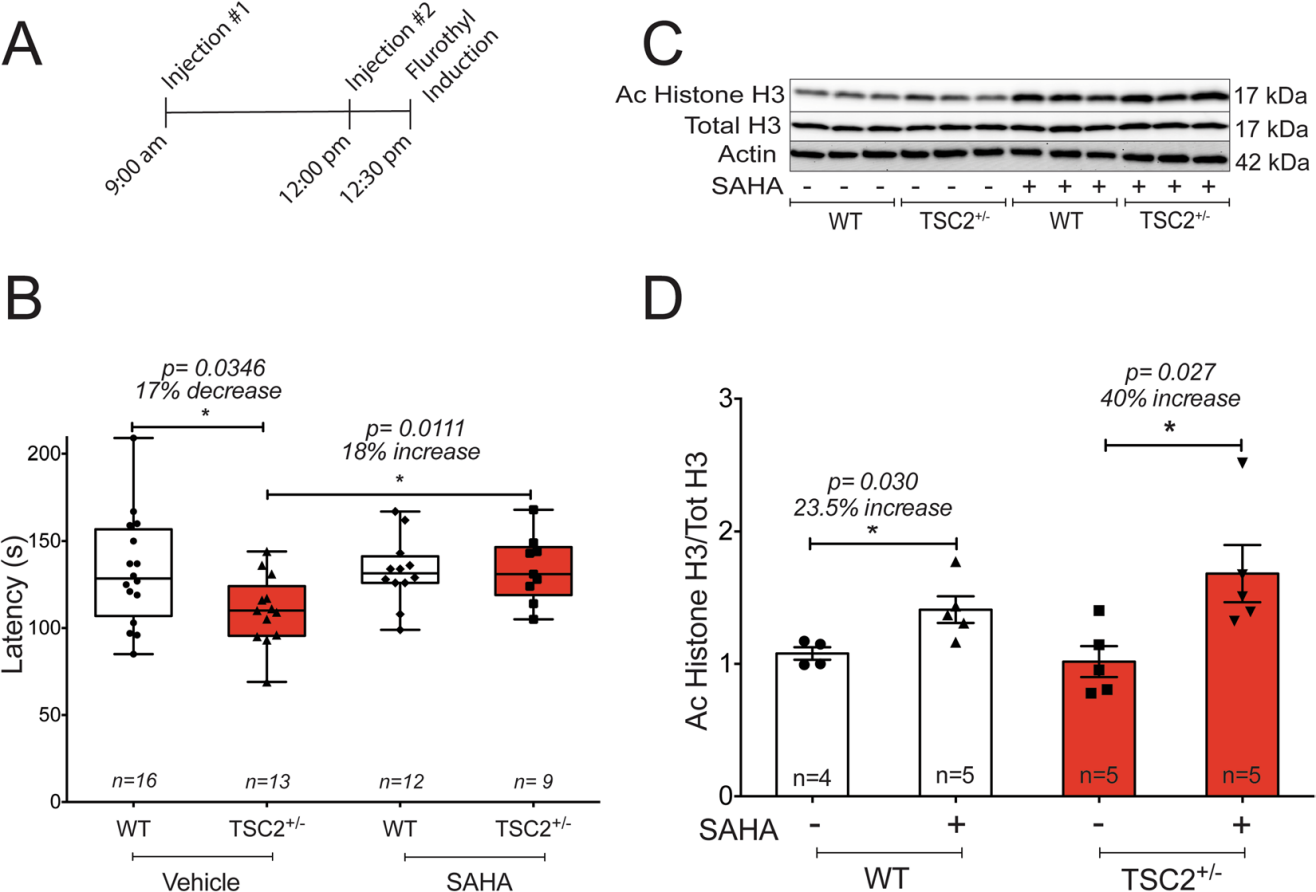
Juvenile TSC2^+/−^ mice exhibit a reduced seizure threshold that is restored to WT-like latency with SAHA. (**A)** A schematic of the injection paradigm used in this study. Juvenile WT and TSC2^+/−^ mice were intraperitoneally injected with either 50 mg/kg SAHA or vehicle (100 mM HPβCD) at the time of injection. Immediately following flurothyl induced seizures, a subset of mice was sacrificed, and whole hippocampal and cortical tissue was collected for post hoc western blot analysis. (**B)** Juvenile TSC2^+/−^ mice exhibit a reduced seizure threshold in response to flurothyl compared to age matched WT mice (Student *t*-test: *p* = 0.0346). Compared to those treated with vehicle (HPβCD), SAHA increased latency to GTCS in juvenile TSC2^+/−^ mice (Student *t*-test: *p* = 0.01) to levels that are indistinguishable from untreated WT mice. Compared to vehicle treated WT mice, WT mice treated with SAHA do not exhibit changes in latency to GTCS. (**C)** Following flurothyl induction, the hippocampi of vehicle or SAHA treated mice were rapidly harvested and processed for western blot analysis. Representative cropped western blot of whole hippocampal lysate extracted from vehicle or SAHA treated mice post flurothyl induction shows a global increase in acetylated histone H3 protein in SAHA treated mice, suggesting SAHA crossed the blood brain barrier and had a physiological effect. Each lane represents lysate from a single animal. An n = 3 per condition is represented in the blot. (**D)** Whole hippocampal extracts acquired from juvenile WT and TSC2^+/−^ mice injected with either SAHA or vehicle were subject to western blot analysis. Quantification of protein levels shows that compared to vehicle treated mice, SAHA treatment increases pan acetyl histone H3 protein levels in the hippocampus of both WT mice (Student *t*-test:*p* = 0.03) and TSC2^+/−^ mice (Student *t*-test: *p* = 0.027).

To test seizure susceptibility, we exposed juvenile WT and TSC2^+/−^ mice to the volatile convulsant, flurothyl (bis (2, 2, 2-trifluoroethyl)). Flurothyl induces generalized tonic clonic seizures (GTCS) in rodents and it acts as a non-competitive antagonist for GABA_A_ receptors. We measured the latency to reach GTCS by placing mice in an enclosed chamber and vaporizing flurothyl until mice experienced a Racine class 5 behavioral seizure, characterized by forelimb clonus accompanied by a complete loss of voluntary motor and postural control^44^. We found that juvenile TSC2^+/−^ mice exhibit a 17% decrease in latency to GTCS compared to age matched littermate WT mice (Fig. 5B), implying a reduced seizure threshold. To determine the impact of HDAC inhibition on seizure threshold, we used the clinically available, blood brain barrier (BBB) permeable and FDA approved class I/II HDACi suberoylanilide hydroxamic acid (SAHA)^45–47^.

Juvenile TSC2^+/−^ mice treated with SAHA (intraperitoneal injection at 50 mg/kg) exhibit a significant increase in latency to reach GTCS compared to vehicle treated TSC2^+/−^ mice (Student *t*-test: *p* = 0.01). In fact, SAHA treated TSC2^+/−^ mice exhibited latency to GTCS that is indistinguishable from both vehicle and SAHA treated juvenile WT mice (Fig. 5B). SAHA treatment did not alter latency to GTCS in juvenile WT mice (Fig. 5B).

Others have reported that SAHA crosses the BBB through systemic administration^45,47^. To confirm that SAHA crosses the BBB in our hands, we performed a western blot analysis of whole hippocampal lysate and probed for global histone acetylation levels. SAHA injected animals exhibit a substantial increase in global acetyl histone H3 (AcH3) levels compared to vehicle injected animals (Fig. 5C,D). These results suggest that class I/II HDACis not only rectifies aberrant synaptic plasticity, but also restores a seizure threshold in TSC2^+/−^ mice to resemble that of age matched WT mice.

## Discussion

The dysregulation of mTORC1 signaling, the subsequent aberrations in protein translation and its role in synaptic plasticity and behavior in TSC rodent models have been the focus of many studies^19,20,22,23^. Herein we provide for the first time, evidence suggesting that the TSC2^+/−^ mouse exhibits an imbalance of HDAC and HAT activity that is driving abnormal synaptic plasticity. In the present study, we show that pharmacological attenuation of HDAC activity in TSC2^+/−^ mice using class I/II HDACis normalizes synaptic plasticity. Further, we describe a reduced seizure threshold in TSC2^+/−^ mice that is suppressed with HDACis to resemble the phenotype observed in WT mice.

Adult TSC2^+/−^ mice exhibit an abnormal acquisition of long lasting LTP with a stimulation paradigm that normally elicits STP in age matched WT mice. Coupled with studies showing that pharmacological attenuation of HDAC activity enhances LTP in WT mice^29,35^, we originally hypothesized that diminished HDAC activity and increased histone acetylation would contribute to the aberrant synaptic plasticity seen in TSC2^+/−^ mice. However, we find that the TSC2^+/−^ mouse hippocampus exhibits a decreased global histone acetylation level. This suggests that there is an imbalance in the ratio of HDAC to HAT activity in TSC2^+/−^ mice.

We proceeded to test whether HDAC inhibitors would reverse synaptic plasticity aberrations in TSC2^+/−^ mice. The adult TSC2^+/−^ mouse hippocampus exhibits an abnormal acquisition of LTP under ‘sub-threshold’ stimulation paradigms. Others have shown that adult TSC2^+/−^ mice display LTP with a single train of 100 Hz, 1 second tetanus^20^. We demonstrate that while a 1 × TBS elicits STP in adult WT hippocampal slices, this stimulation is enough to induce long lasting LTP in adult TSC2^+/−^ mice. The aberrant capture of LTP under ‘sub-threshold’ stimulation paradigms has been suggested to be a potential cellular/circuit correlate for the impaired hippocampal dependent learning and perseverative behavior characterized in rodent models of TSC^20^. Using TSA to pharmacologically attenuate heightened HDAC activity in adult TSC2^+/−^ hippocampal slices, we are able to restore a normal STP response to the 1 × TBS stimulation paradigm. To our knowledge, this is the first study suggesting that aberrant chromatin structure may contribute to altered synaptic plasticity in the TSC2^+/−^ mice.

Interestingly, acute hippocampal slices from adult WT mice provoked with either a single 100 Hz stimulation^29,35^ or 1 × TBS exhibit enhanced LTP in the presence of TSA. In contrast to the 1 × TBS phenotype exhibited by WT slices treated with TSA, attenuating HDAC function in acute hippocampal slices from adult TSC2^+/−^ mice yields STP. These paradoxical findings hint at fundamentally different wiring of chromatin modifying machinery and plasticity mechanisms in the WT and TSC2^+/−^ hippocampus. Future experiments will be directed towards understanding how HDAC inhibition induces opposing plasticity phenotypes in the WT and TSC2^+/−^ hippocampus.

Juvenile TSC2^+/−^ mice (p21–24) exhibit a reduced mGluR-LTD magnitude^19,24^. The mechanistic and behavioral consequences of a reduced mGluR-LTD in the hippocampus are still under debate, but it has been suggested that it could influence a preference for LTP and promote a hyperexcitable neural network^40^. Inhibiting mTORC1 activity with rapamycin has been shown to restore a normal mGluR-LTD in juvenile TSC2^+/−^ mice^19,24^. Intriguingly, the juvenile WT hippocampal mGluR-LTD, though dependent on protein synthesis, functions independently of mTORC1 signaling^19^. Here, we show that attenuating HDAC function increases the mGluR-LTD magnitude in juvenile TSC2^+/−^ mice to match that observed in WT mice.

Wild type mice exhibit an mTORC1 independent mGluR-LTD as juveniles that transitions to an mTORC1 dependent LTD as adults^19,24^. Interestingly, juvenile TSC2^+/−^ mice switch from displaying an mGluR-LTD that is inhibited by mTORC1 to exhibiting an mGluR-LTD that is not reliant on mTORC1 in adulthood^24^. We have shown that this phenotype is driven by increased MAPK signaling as attenuation of ERK1/2 signaling in the TSC2^+/−^ brain restores WT-like, mTORC1 dependent mGluR-LTD^24^. In this study, we use two structurally distinct class I/II HDACis (TSA and VPA) to demonstrate that we can restore rapamycin sensitive, mTORC1 dependent mGluR-LTD in the adult TSC2^+/−^ mice. In summary, we show that pharmacological inhibition of HDAC activity normalizes the altered synaptic plasticity exhibited by TSC2^+/−^ mice.

ERK1/2 is an activity dependent kinase that positively regulates mTORC1^48^. We have shown that TSC2^+/−^ mice exhibit increased hippocampal ERK1/2 activity. Attenuation of hyperactive ERK1/2 restores normal mGluR-LTD in TSC2^+/−^ mice^24^. This result mirrors the observation we make with HDAC inhibition in the current study. The similarity in the synaptic plasticity output suggests a possible link between potentially enhanced HDAC function and increased ERK1/2 activity in the TSC2^+/−^ mice. Future studies will be directed towards understanding one of three possibilities: (1) altered HDAC/HAT activity in TSC2^+/−^ mice is driving ERK1/2 disinhibition; (2) ERK1/2 upregulation is contributing to enhanced HDAC function, or (3) HDACs and ERK1/2 are functioning independently of one another.

Over 85% of TSC patients exhibit seizures within the first year of life^9,10,43^. The early onset for epilepsy predisposes TSC patients to cognitive deficits that persist beyond childhood. In accordance with the young age of seizure onset in TSC patients, we find that juvenile TSC2^+/−^ mice (p18–p21) exhibit a decreased seizure threshold compared to age matched WT mice. This is the first report of a seizure phenotype in the TSC2^+/−^ mouse model, though it is consistent with a seizure phenotype reported in TSC1^+/−^ juvenile mice^49^. We go on to demonstrate that attenuating class I/II HDACs using SAHA restores a seizure threshold indistinguishable from littermate WT mice.

The decreased seizure threshold in juvenile TSC2^+/−^ mice can potentially induce long term changes in neural connectivity that persist beyond development to influence and contribute to the abnormal synaptic plasticity that we and others report in the adult TSC2^+/−^ mice. This concept is directly applicable to human TSC patients, in that altered network excitability early in life could influence disease progression in adulthood. It would be interesting to test whether attenuating HDAC function in TSC2^+/−^ mice for a period of time during development could possibly normalize the altered synaptic plasticity that adult TSC2^+/−^ mice exhibit.

The HDACis used in this study broadly target both class I and II HDACs. HDACs 1, 2, 3, 4 and 6 differentially regulate learning, memory and synaptic plasticity^29–33,50^. Thus, there may also be distinct regulation, recruitment and function of HDACs in the TSC brain. Because we used pan HDACis in this study, we cannot definitively identify which HDAC(s) contributes to the neurological manifestations in the TSC brain. Knockdown studies will help determine which particular HDAC, or group of HDACs, is hyperactive in the TSC brain.

We bath applied either TSA or VPA for at least one hour prior to mGluR-LTD or 1 × TBS induction (see Methods section). The timing of drug application was determined through a series of pilot experiments in which we found that synaptic plasticity in TSC2^+/−^ hippocampal slices only mirrored a WT response when the HDACis were present for at least one hour prior to induction. Studies by others demonstrates that bath application of HDACis for as short as 30 minutes^38^ or even 20 minutes^29,35^ elicits an enhanced LTP in adult WT hippocampal slices. These studies show that a relatively short bath application of HDACis is enough to exert an effect on synaptic plasticity.

HDACs cannot directly bind to DNA to regulate gene expression. HDACs exist as a part of multiprotein co-repressor complexes that are recruited by DNA bound transcription factors. If increased HDAC function in the TSC2^+/−^ mouse model is primarily working to reduce global histone acetylation levels and repress target gene expression, identifying which transcription factor(s) are preferentially recruiting HDACs and then exploring the genes that may be differentially regulated with enhanced HDAC activity can help with developing therapeutic targets for TSC patients.

It is formally possible that the restorative effects of HDAC inhibition on the synaptic plasticity deficits in TSC2^+/−^ mice may be functioning through non-histone targets outside of the nucleus. Future work will determine whether inhibiting class I HDACs to ameliorate the neurological manifestations in TSC works via transcriptional or non-transcriptional targets.

Our current study does not explore the mechanism that contributes to the global reduction in histone acetylation levels in TSC2^+/−^ mice. While we observe a restoration of normal neurological function in the TSC2^+/−^ mice by attenuating HDAC activity, this does not eliminate a potential for diminished HAT activity which would manifest in the reduced global histone acetylation that we observe in the TSC2^+/−^ mouse brain. An imbalance in the ratio of available HDACs in comparison to acetylated targets may favor a global hypoacetylation in TSC2^+/−^ brains. Consequently, our use of HDAC inhibitors to restore normal synaptic plasticity and increase seizure threshold to WT levels may be working by resetting the ratio of available HDACs and acetylated targets. Future studies will be directed towards exploring whether TSC2^+/−^ brains exhibit decreased HAT activity that may be driving the altered neuroplasticity and reduced seizure threshold phenotypes we observe in this TSC mouse model.

Approximately 50% of TSC patients exhibit ASD^9^ and it should be noted that recent reports reveal a role for increased HDAC activity in ASD-like social deficits in *Shank3*-deficient mice^51,52^ and in the BTBR T + tf/J (BTBR) mouse model of autism^53^. These studies extend the potential therapeutic benefits of HDACis in treating TSC patients.

In summary, this is the first study suggesting that TSC synaptic plasticity and seizure propensity are regulated by altered chromatin structure. Data presented herein suggest that HDACis may provide an alternative therapeutic target for ameliorating the neurological symptoms manifest in TSC.

## Methods

### Mice

All animal procedures were performed with the approval of the University of Wisconsin-Madison School of Medicine and Public Health Institutional Animal Care and Use Committee and according to national guidelines and policies. Male littermate WT and TSC2^+/−^ (C57BL/6 strain) mice were used for all experiments in this study. Mice were kept in standard housing cages and kept on a typical 12 hours light/12 hours dark cycle (lights on at 6:00 A.M). Mice had *ad libitum* access to water and food.

### Drugs

(*S*)-3, 5- dihydroxyphenylglycine (*S*-DHPG) and suberanilohydroxamic acid (SAHA) were purchased from Tocris Biosciences. Trichostatin A (TSA), valproic acid (VPA), rapamycin, and bis (2, 2, 2-trifluoroethyl) ether (flurothyl) were purchased from Sigma-Aldrich. DHPG and VPA were solubilized in MilliQ water. Stock aliquots of TSA and rapamycin were solubilized in dimethyl sulfoxide (DMSO). To account for the known difficulty in solubilizing SAHA, we followed the *in vivo* preparation methods previously published by Hockly, E. *et al*.^45,47^. 2-hydroxypropyl-β-cyclodextrin powder (HPβCD) was purchased from Acros Organics. SAHA was dissolved in 100 mM HPβCD by boiling for 5 minutes. The solution was slowly cooled at room temperature prior to administering the drug intraperitoneally to mice.

### Electrophysiology

Electrophysiology was performed on acute hippocampal slices prepared from juvenile (p20–p24) and adult (6–8 weeks old) littermate wild type (WT) and TSC2^+/−^ mice (C57BL/6 strain). Hippocampal slice preparations were performed at the same time for every experiment. After decapitation, the brain was rapidly extracted and placed in to a frozen slurry of cutting solution (CS; 0.6 mM sodium ascorbate, 3 mM KCl, 1.25 mM NaH_2_PO_4_, 60 mM NaCl, 28 mM NaHCO_3_, 7 mM MgCl_2_, 0.5 mM CaCl_2_, 5 mM D-glucose, and 110 mM sucrose). Horizontal sections of the hippocampus (400 µm) were made using a Vibratome while submerged in the CS slurry. After sectioning, slices recovered at room temperature for 45 minutes in a solution containing 50% CS and 50% artificial cerebrospinal fluid (ACSF; 2.5 mM KCl, 1.25 mM NaH_2_PO_4_, 125 mM NaCl, 25 mM NaHCO_3_, 1 mM MgCl_2_, 2 mM CaCl_2_, and 25 mM D-glucose). Slices were then placed in a beaker containing 100% ACSF at room temperature for an additional 45 minutes. After recovery, slices were transferred to an interface chamber (Fine Science Tools, Foster City, CA). Hippocampal slices were equilibrated on the recording rigs for 2 hours while being perfused with ACSF warmed to 32 °C (TC-324B, Warner Instrument Corporation, Hamden, CT) at a rate of 1.5 mL/minute using a peristaltic pump. All solutions used in the presence of live tissue were constantly carb-oxygenated (95% O_2_/5% CO_2_).

Bipolar stimulating electrodes were made using isonel enameled platinum-tungsten wire (92:8 Pt:Y; Sigmund Cohn Corporation, Mt. Vernon, NY) and placed on the Schaeffer collateral axon bundles extending from the CA3 to CA1. Recording electrodes were made from single barrel borosilicate capillary glass pipettes with microfilament (A-M Systems). The electrodes were filled with ACSF (4–5 MΩ) and placed on the CA1 stratum radiatum. Baseline synaptic transmission was evaluated by measuring input:output relationship (0.5 V-20 V, 25 nA-2 µA, using A-M Systems model 2200 stimulus isolator). Subsequent stimulations for the slice were set at 50% the maximum field excitatory post synaptic potential (fEPSP) slope elicited from the input:output relationship. Paired pulse facilitation (PPF) were conducted as a measure of pre-synaptic transmission and as a measure of slice health. For the PPF paradigm, Slices were stimulated with two pulses, an initial stimulus followed by subsequent stimulations separated by increasing interstimulation intervals. Pre-synaptic mediated facilitation was analyzed by measuring the slopes of the evoked fEPSPs and comparing each response as a function of the initial evoked fEPSP.

Slices were stimulated every 20 seconds, and the average of 2 minute sweeps were used to generate a single data point. The fEPSPs were amplified (A-M systems model 1800) and digitized (100 kHz, Digidata 1322B, Molecular Devices) prior to being analyzed (pClamp, Molecular Devices). Graphical representations of the data were generated by measuring post induction fEPSP slopes and normalizing them to the average fEPSP slope at baseline (60 minutes prior to LTP or LTD induction). Slices with unstable baselines (>10% deviation from across the baseline) were not used in final data analysis.

### LTP Induction

We used a single theta burst stimulation (1 × TBS) to elicit STP in acute hippocampal slices. A 1 × TBS paradigm consisted of 1 train of 10 bursts. Each burst had 4 stimulations at 100 Hz, each separated by 200 milliseconds. In experiments with TSA, baseline recordings were first monitored without drug for 30 minutes followed by an additional hour with TSA 1.65 µM. Slices were stimulated with 1 × TBS after an hour incubation in TSA. Once introduced to the slices, TSA was left on for the duration of the experiment.

### LTD Induction

mGluR-LTD was induced using prolonged incubation of *S*-DHPG (50 µM for 10 minutes). We recorded baseline response for 30 minutes prior to drug application. Where applicable, hippocampal slices were incubated in TSA (1.65 µM) or VPA (250 nM) for 60 minutes prior to introduction of rapamycin (20 nM for 20 minutes). Slices were then incubated with *S*-DHPG (50 µM for 10 minutes) in the presence of rapamycin, TSA or VPA. For appropriate experiments, rapamycin treatment continued for 10 minutes after *S*-DHPG was removed from the recording chamber. Where applicable, TSA or VPA remained present for the duration of the experiment. Time “0” for mGluR-LTD was set at the point when the evoked fEPSP responses fell below the average baseline response by greater than 10%.

### Tissue homogenization and western blotting

Acute hippocampal slices were harvested in to Eppendorf tubes that were pre-chilled on dry ice (one hippocampal slice per tube) and immediately flash frozen in liquid nitrogen following drug incubations. Slices were lysed using radioimmunoprecipitation assay (RIPA) buffer (50 mM Tris [pH 7.5], 150 mM NaCl, 1% nonidet P-40, 0.5% sodium deoxycholate, 0.1% SDS; made in Milli-Q water) in the presence of phosphatase inhibitors (10 mM NaF, 2 mM Na Vanadate, 4 mM Na pyrophosphate, and 10 mM Na β-glycerophosphate; made in RIPA buffer) and protease inhibitors (used at 1:100; from Sigma Aldrich). The lysates were centrifuged at 12,000 RPM for 30 minutes at 4 °C. Protein was quantified using the DC Protein Assay (Bio-Rad, Hercules, CA). Sample concentrations were standardized with 5X loading buffer (0.25 M Tris pH 6.8, 10% SDS, 50% glycerol, 0.25% bromophenol blue, and 5% β-mercaptoethanol) and RIPA. Samples were then boiled at 95 °C for 8 minutes.

Protein lysates were loaded onto 4–15% precast gradient gels (BioRad Laboratories) at 15 µg per lane and run (35 mA/gel, ~150 V) in the presence of standard electrophoresis buffer (100 mM Tris, 1.5 M glycine, and 0.1% SDS). Proteins were transferred onto polyvinyl difluoride (PVDF) membrane (Merck-Millipore) in standard transfer buffer (20 mM Tris, 1.5 M glycine, and 20% methanol) at 4 °C. The membrane was then blocked for one hour in 5% milk solution made up in low salt, Tris-based salt solution with Tween-20 (low salt TBST; 20 mM Tris pH 7.6, 150 mM NaCl, 0.1% Tween-20). All blots were incubated in primary antibody overnight. All antibodies were diluted in 5% milk dissolved in low salt TBST. All antibodies were used at 1:1000 unless otherwise noted. The following antibodies were purchased from Cell Signaling Technology: H3K9Ac, H3K27Ac, Total Histone H3. Anti-AcHistoneH3 was purchased from Millipore. Actin was purchased from MP Biomedicals and used at a 1:10,000 dilution.

After removal of primary antibody, blots were washed (3 times, at 10 minutes per wash) with low salt TBST prior to the addition of horseradish peroxidase-conjugated secondary antibodies (either goat anti-mouse IgG or goat anti-rabbit IgG; 1:10,000 in 5% milkfat + low salt TBST; Santa Cruz Biotechnology, Santa Cruz, CA). Protein bands were detected using either SuperSignal West FEMTO ECL reagent (Pierce Biochem) or ECL (ThermoScientific). Images were captured using a UVP ChemiDoc-it Imaging System and the VisionWorks software was used to quantify protein bands.

### Flurothyl

The flurothyl seizure inductions were conducted on juvenile (p18–p21) WT and TSC2^+/−^ mice using 100% bis (2, 2, 2- trifluoroethyl. Mice were placed in an airtight 10 L Plexiglas chamber (8.5″ × 10.75″ × 6.75″). Flurothyl was infused into the chamber through a peristaltic pump (40 µL/min) onto a Whatman filter paper located at the top of the chamber. The latency to a generalized tonic clonic seizure (GTCS) was measured from the start of flurothyl infusion into the chamber. The GTCS state was defined as a complete loss of motor and postural control. Once the mouse reached GTCS, flurothyl infusion was quickly terminated and the mouse was rapidly removed from the chamber. All flurothyl experiments were video recorded for behavioral assessment by a blinded reviewer. For biochemistry experiments, mice were sacrificed within 2 minutes of exiting the flurothyl chamber. Tissue was rapidly extracted, put into dry ice chilled Eppendorf tubes, and flash frozen in liquid nitrogen. The tissue was stored at −80 °C until processed for biochemistry. The chamber was aired out thoroughly and cleaned with 70% ethanol between inductions.

Injection of the vehicle (100 mM HPβCD) alone did not produce an adverse reaction in the mice as determined through behavioral assessments (i.e. irregular grooming, orbital tightening, aggression, lethargy and/or hunched posture).

Animals were injected intraperitoneally with either a vehicle solution (100 mM HPβCD) or SAHA (50 mg/kg) twice on the day of testing. Animals were injected at 9 A.M. on the morning of testing, and injected again with the same treatment exactly 3 hours later. Post injection, animals were allowed to recover in a quiet room for at least 30 minutes prior to flurothyl induction.

Personnel blinded to mouse genotypes performed all drug treatments, flurothyl testing and post induction latency scoring.

### Statistical analysis

For all tests, *p* < 0.05 was considered statistically significant. All data is represented as the mean +/− standard error of the mean (SEM). All data was analyzed and graphical representations were created through Prism 6 (Graphpad Software, La Jolla, CA).

#### Electrophysiology

Data was analyzed by conducting two-way ANOVAs with repeated measures (mixed model). Multiple comparisons were corrected using the Bonferroni test. For statistics, the “n” was treated as individual slices obtained from a group of mice for a given condition.

#### Western blot

Protein bands from western blots were quantified using UVP VisionWorks software. Western blot data was analyzed by conducting unpaired, parametric two-tailed Student *t* tests.

#### Flurothyl

The latency to GTCS was analyzed between genotypes and between treatment conditions using unpaired, parametric two-tailed Student *t* tests. Personnel blinded to genotypes and treatment groups scored all flurothyl induction videos.

## Supplementary information

Supplemental Information

## Data Availability

The datasets generated and analyzed during the course of the current study are available from the corresponding author upon reasonable request.
