# Supplementary material for: Histone deacetylase inhibitors restore normal hippocampal synaptic plasticity and seizure threshold in a mouse model of Tuberous Sclerosis Complex
Source: Sci Rep. 2019 Mar 27;9:5266. doi: 10.1038/s41598-019-41744-7 (PMC6437206; doi:10.1038/s41598-019-41744-7)
Supplement: Supplementary file 1 — Supplemental Information [file 41598_2019_41744_MOESM1_ESM.pdf]

Histone deacetylase inhibitors restore normal hippocampal synaptic plasticity and seizure threshold in a mouse model of Tuberous Sclerosis Complex.

Trina Basu[1,2], Kenneth J. O'Riordan[5], Barry A. Schoenike[1], Nadia N. Khan[1,3], Eli P. Wallace[1,4], Genesis Rodriguez[1], Rama K. Maganti[5], Avtar Roopra[1,2,3*]

1 Department of Neuroscience, University of Wisconsin-Madison, Madison, Wisconsin, United States of America

2 Neuroscience Training Program, University of Wisconsin-Madison, Madison, Wisconsin, United States of America

3 Graduate Program in Cellular and Molecular Biology, University of Wisconsin-Madison, Madison, Wisconsin, United States of America

4 Cellular and Molecular Pathology Graduate Program, University of Wisconsin-Madison, Madison, Wisconsin, United States of America

5 Department of Neurology, University of Wisconsin-Madison, Madison, Wisconsin, United States of America

*Corresponding author; email: asroopra@wisc.edu

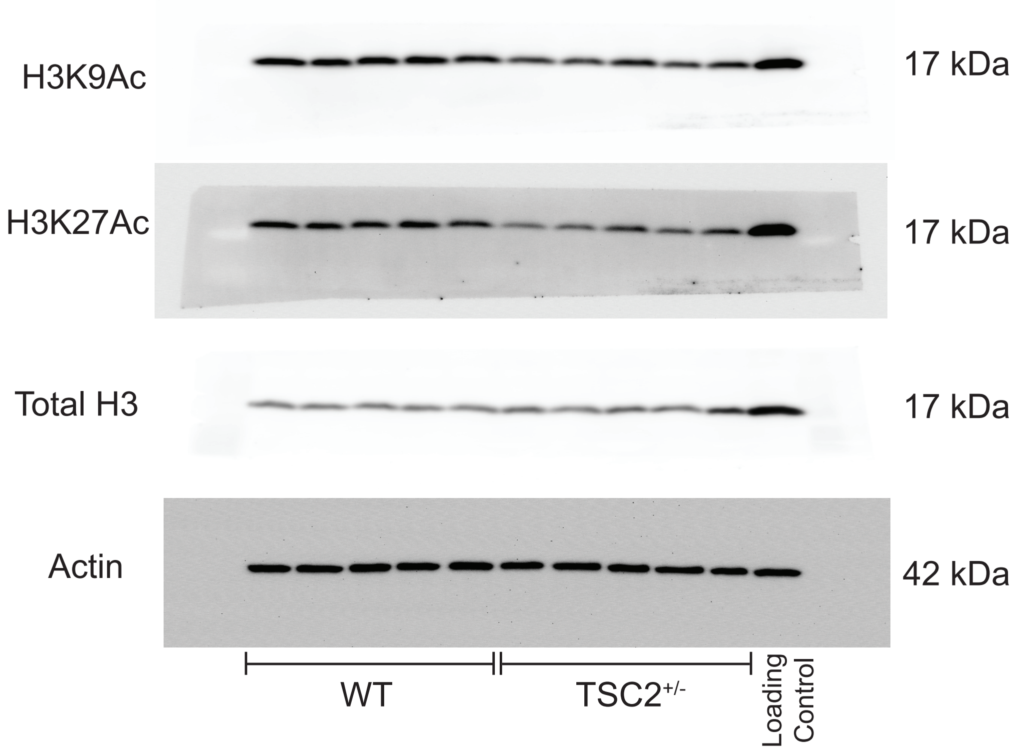

<u>Supplemental Figure 1</u>: **TSC2$^{+/-}$ mouse hippocampi exhibit decreased H3K9 and H3K27 acetylation levels.** These representative western blots are the whole, uncropped versions of the blots shown in Figure 1A of the manuscript. The samples depicted here are from adult wildtype (WT) and TSC2$^{+/-}$ hippocampal slices. Each lane represents a single slice (n= 5 per genotype) harvested after incubation in artificial cerebrospinal fluid (ACSF) for 4 hours. The last lane represents a common WT hippocampal lysate control that was loaded across gels to normalize protein quantifications of samples run on separate blots. All samples were lysed using radioimmunoprecipitation assay (RIPA) buffer. The blots representing H3K9Ac and H3K27Ac were imaged using SuperSignal™ West Femto Maximum Sensitivity Substrate from Thermo Scientific™ and exposed for 30 seconds. The blots representing

Total H3 and Actin were imaged using Enhanced Chemilumiscent Western Blotting reagents from GE Healthcare and were exposed for 1 minute and 30 seconds. The brightness (50), contrast (50) and gamma (1.0) signals were the same for each image generated for this figure.

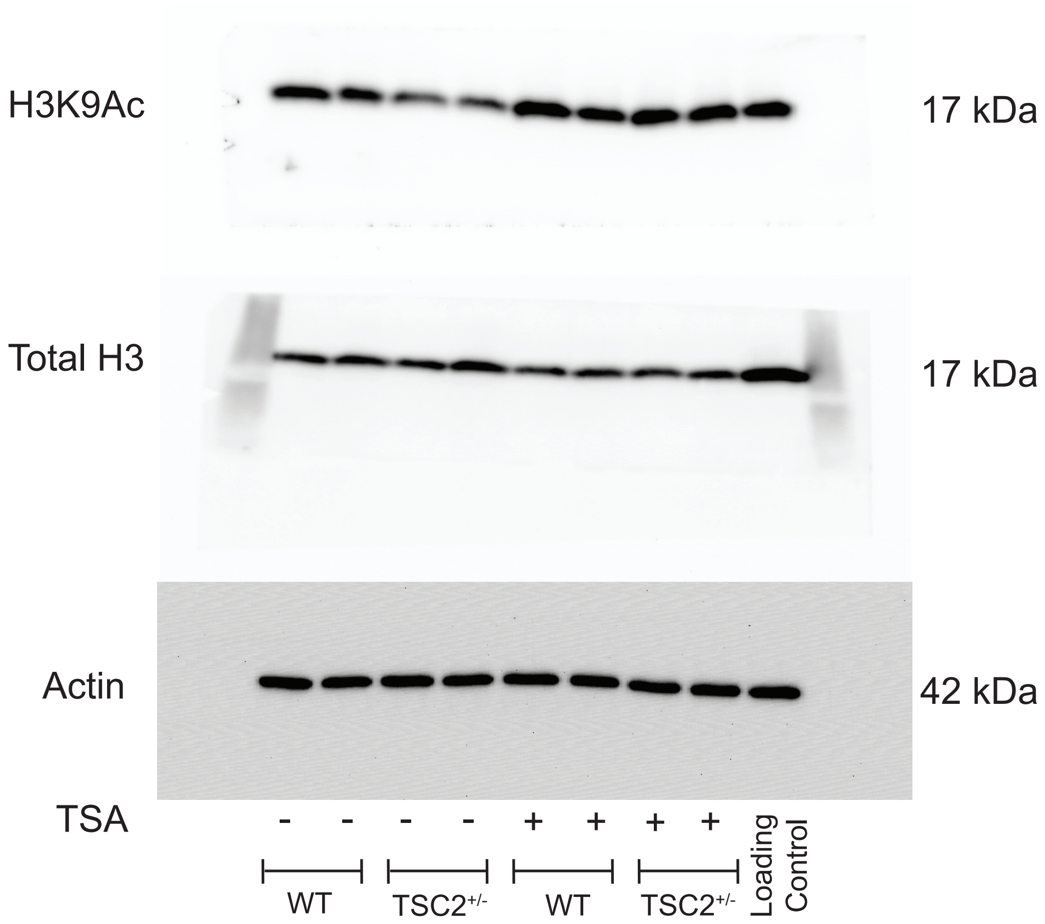

<u>Supplemental Figure 2</u>: TSA increases H3K9Ac levels in both WT and TSC2$^{+/-}$ hippocampal extracts. These representative western blots are the whole, uncropped versions of the blots shown in Figure 1B of the manuscript. The data shown here are hippocampal slice lysates from adult WT and TSC2$^{+/-}$ mice. Slices were treated with or without Trichostatin A (TSA; 1.65 μM) for 4 hours and rapidly harvested following drug treatment. The last lane represents a common WT hippocampal lysate control that was loaded across gels to normalize protein quantifications of samples run on separate blots. All samples were lysed using RIPA buffer. All the blots shown in this figure were imaged using Enhanced Chemilumiscent Western Blotting reagents from GE

Healthcare and were exposed for 1 minute and 30 seconds. The brightness (50), contrast (50) and gamma (1.0) signals were the same for each image generated for this figure.

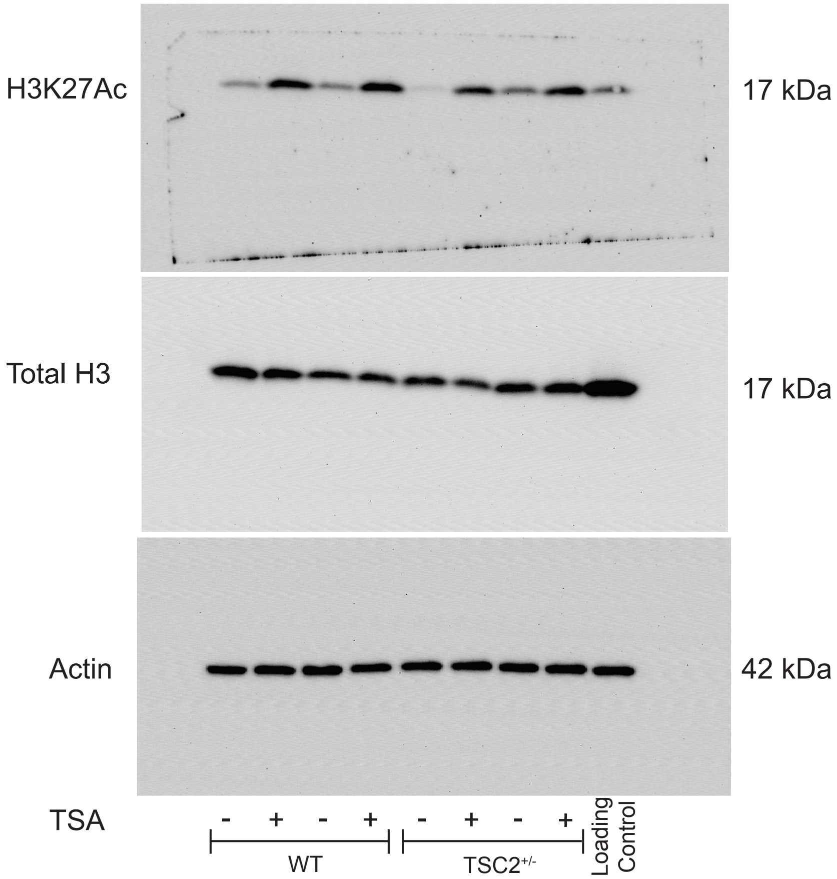

<u>Supplemental Figure 3</u>: TSA increases H3K27Ac levels in both WT and TSC2$^{+/-}$ hippocampal extracts. These representative western blots are the whole, uncropped versions of the blots shown in Figure 1C of the manuscript. Shown here are hippocampal slice lysates extracted from adult WT and TSC2$^{+/-}$ mice. Slices were treated with or without TSA (1.65 μM) for 4 hours and rapidly harvested following drug treatment. The last lane represents a common WT hippocampal lysate control that was loaded across gels to normalize protein quantifications of samples run on separate blots. All samples were lysed using RIPA buffer. All the blots shown in this figure were imaged using Enhanced Chemilumiscent Western Blotting reagents from GE

Healthcare and were exposed for 1 minute and 30 seconds. The brightness (50), contrast (50) and gamma (1.0) signals were the same for each image generated for this figure.

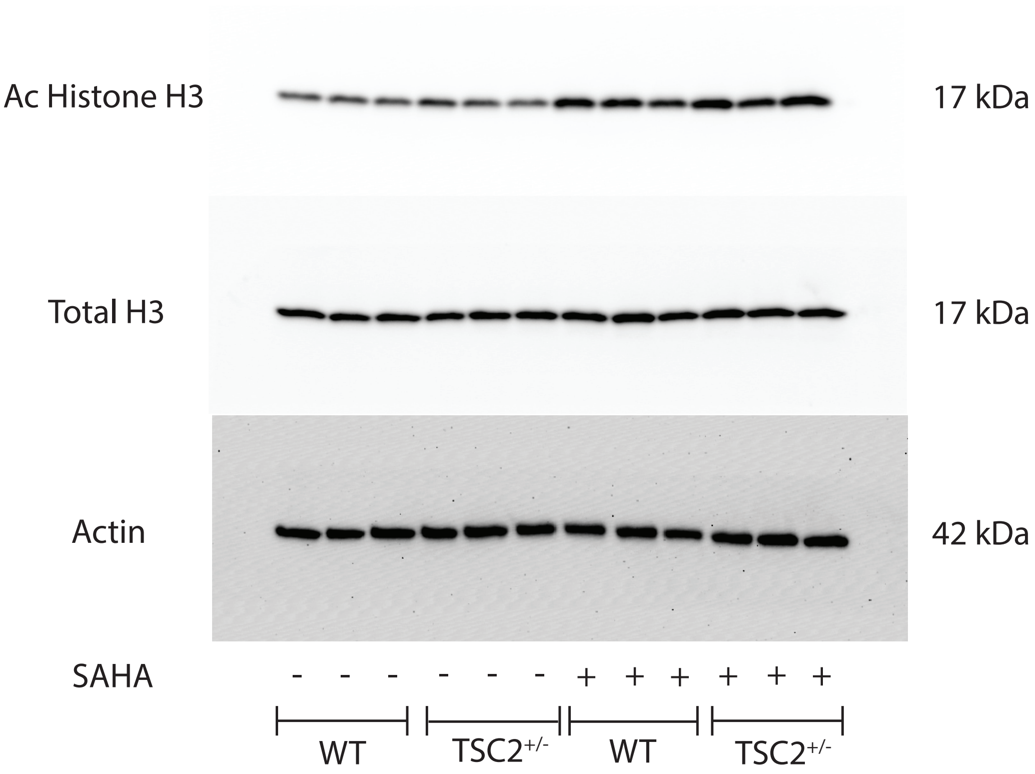

<u>Supplemental Figure 4</u>: Systemically injected SAHA crosses the blood brain barrier. These representative western blots are the whole, uncropped versions of the blots shown in Figure 5C of the manuscript. The samples are from whole hippocampal lysates extracted from juvenile (p18-p21) mice. Mice were injected with suberolanilide hydroxamic acid (SAHA; 50 mg/kg) or vehicle (2-hydroxypropyl-ß-cyclodextrin; HPßCD) for three days prior to flurothyl test day. On the day of flurothyl testing (the third day of either SAHA or HPßCD injection), animals were injected at 3 hours and again at 30 minutes prior to flurothyl induction. The hippocampus was rapidly extracted after flurothyl induction and flash frozen in liquid nitrogen until processing for western blot analysis. All samples were lysed using RIPA buffer. All the blots shown in this figure were imaged using Enhanced Chemilumiscent Western Blotting reagents from GE

Healthcare and were exposed for 1 minute and 30 seconds. The brightness (50), contrast (50) and gamma (1.0) signals were the same for each image generated for this figure.

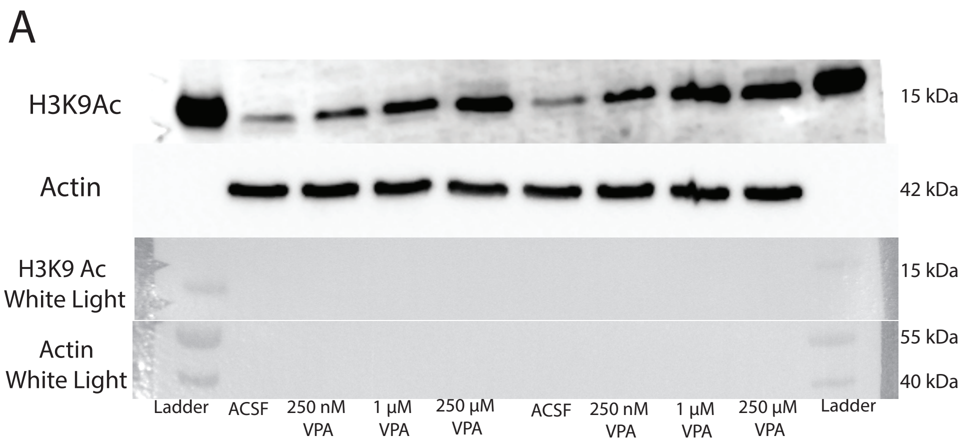

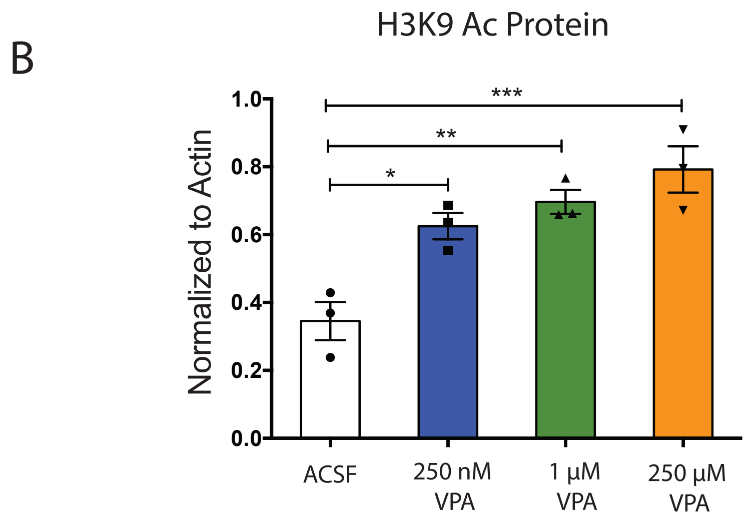

<u>Supplemental Figure 5</u>: **Inhibiting HDACs with 250 nM valproic acid (VPA) increases H3K9 acetylation.** A) This is a representative, uncropped western blot of acutely harvested hippocampal slices from adult WT mice. The first 4 lanes represent slices harvested from one mouse and the last 4 lanes represent slices from another mouse. The slices were all treated with varying doses of VPA in ACSF for 1 hour on an interface chamber that was constantly carboxygenated with 95%/5% O_2/CO_2 mix. Control slices that were not treated with VPA remained in ACSF for 1 hour prior to

harvest. Slices were flash frozen in liquid nitrogen and stored in a -80 C freezer until they were processed for western blot analysis. B) Quantification of H3K9Ac protein levels in slices treated with or without varying doses of VPA. An n= 3 slices from 3 mice per condition was analyzed via western blot analysis. (One-way ANOVA; $F(3,8)= 13.91$, $p= 0.0015$. Tukey’s post hoc multiple comparisons test was used to compare means between conditions).
